# Technical performance analysis of different types of spirometers

**DOI:** 10.1186/s12890-021-01752-8

**Published:** 2022-01-05

**Authors:** Zhongping Wu, Ruibo Huang, Liping Zhong, Yi Gao, Jinping Zheng

**Affiliations:** National Center for Respiratory Medicine, State Key Laboratory of Respiratory Disease, National Clinical Research Center for Respiratory Disease, Guangzhou Institute of Respiratory Health, First Affiliated Hospital of Guangzhou Medical University, Yanjiang Road 151, Guangzhou, 510120 Guangdong People’s Republic of China

**Keywords:** Spirometer, Standard flow/volume simulator, Quality control, ISO 26782:2009

## Abstract

**Background:**

The spirometer is an important element in lung function examinations, and its accuracy is directly related to the accuracy of the results of these examinations and to the diagnosis and treatment of diseases. Our aim was to conduct a performance analysis of the detection techniques of differential pressure and ultrasonic portable spirometers commonly used in China.

**Methods:**

A standard flow/volume simulator was used to analyze the performance (accuracy, repeatability, linearity, impedance, and so on) of portable spirometers, 4 imported and 6 domestic, based on 13 curves generated by different air sources in the ISO 26782:2009 standard. A Bland–Altman diagram was used to evaluate the consistency between the values measured by the spirometers and the simulator.

**Results:**

The pass rates for accuracy, repeatability, linearity, and impedance for the 10 different portable spirometers were 50%, 100%, 70%, and 70%, respectively. Only 30% (3/10) of the spirometers—2 domestic and 1 imported—met all standards of quality and performance evaluation, while the rest were partially up to standard. In the consistency evaluation, only 3 spirometers were within both the consistency standard range and the acceptability range.

**Conclusion:**

The quality and performance of different types of portable spirometers commonly used in the clinic differ. The use of a standard flow/volume simulator is helpful for the standard evaluation of the technical performance of spirometers.

**Supplementary Information:**

The online version contains supplementary material available at 10.1186/s12890-021-01752-8.

## Introduction

A spirometer is a medical device used to record physiological lung volume within the range of vital capacity [1]. The use of a spirometer to assess the volume of air that a patient can exhale within a certain period of time after maximum inspiration is helpful in diagnosing restrictive lung disease, airway obstruction and other lung diseases. Technological and scientific advancements have helped evolve the spirometer from fixed, volume-based devices to portable, flow-based devices that can be transported easily [2]; to date, more than a dozen kinds of spirometers from different brands, both domestic and foreign, are available. However, whether the performance of different brands of spirometer is consistent and meets technical standards remain to be verified.

At present, quality control is receiving increasing attention in the development of pulmonary function tests at home and abroad, serving as the lifeline of lung function examinations [3]. Most of the domestic testing and calibration of the accuracy of spirometers is performed through self-tests of the instrument and the use of standard calibration cylinders. Although this method is simple and convenient, it is unable to measure forced expiratory volume in the first second (FEV_1_) and other indicators because there is only a single volume and flow rate that cannot be controlled accurately, which is not sufficient for quality control of spirometer. In 2009, the International Organization for Standardization (ISO) published “Anesthetic and respiratory equipment—Spirometers intended for the measurement of time forced expired volumes in humans (ISO 26782:2009)”, in which 13 different volume-time curves were formulated, corresponding to different values of FEV_1_, forced expiratory volume in 6 s (FEV_6_), forced vital capacity (FVC) and other indicators [4]. Different air sources generated by a simulator can be used to test spirometers, and the resulting test value can be compared with the actual value of the simulator to analyze the performance of the instruments. In this study, we used a standard simulator to determine the performance of portable spirometers commonly used in our country.

## Objects and methods

### Object

The research objects were 10 portable or hand-held flow spirometers currently used in the clinic in China, including 3 ultrasonic spirometers (Easy on-PC, NDD, Switzerland; XiaoCool, ANCOOL, China; Powercube-Body, GANSHORN, Germany) and 7 differential pressure spirometers (PC-10, CHEST, Japan; HCY-02, SONMOL, China; X1, XEEK, China; MasterScope, Jaeger, Germany; PF680, U-BREATH, China; LA104, MEHOW, China; A1, BreathHome, China). Four of these spirometers were imported and six were domestic, all of which were brand new instruments. In subsequent tests, numbers 1–10 were used for description instead of these spirometers.

### Experimental equipment

A standard flow/volume simulator (Model 1120, Hans Rudolph, USA), hereinafter referred to as the simulator, was used for testing. The simulator is a standard gas source that serves as an accurate instrument for simulating the basic movement patterns of the human lung.

### Test waveform

**Fig. 1:**
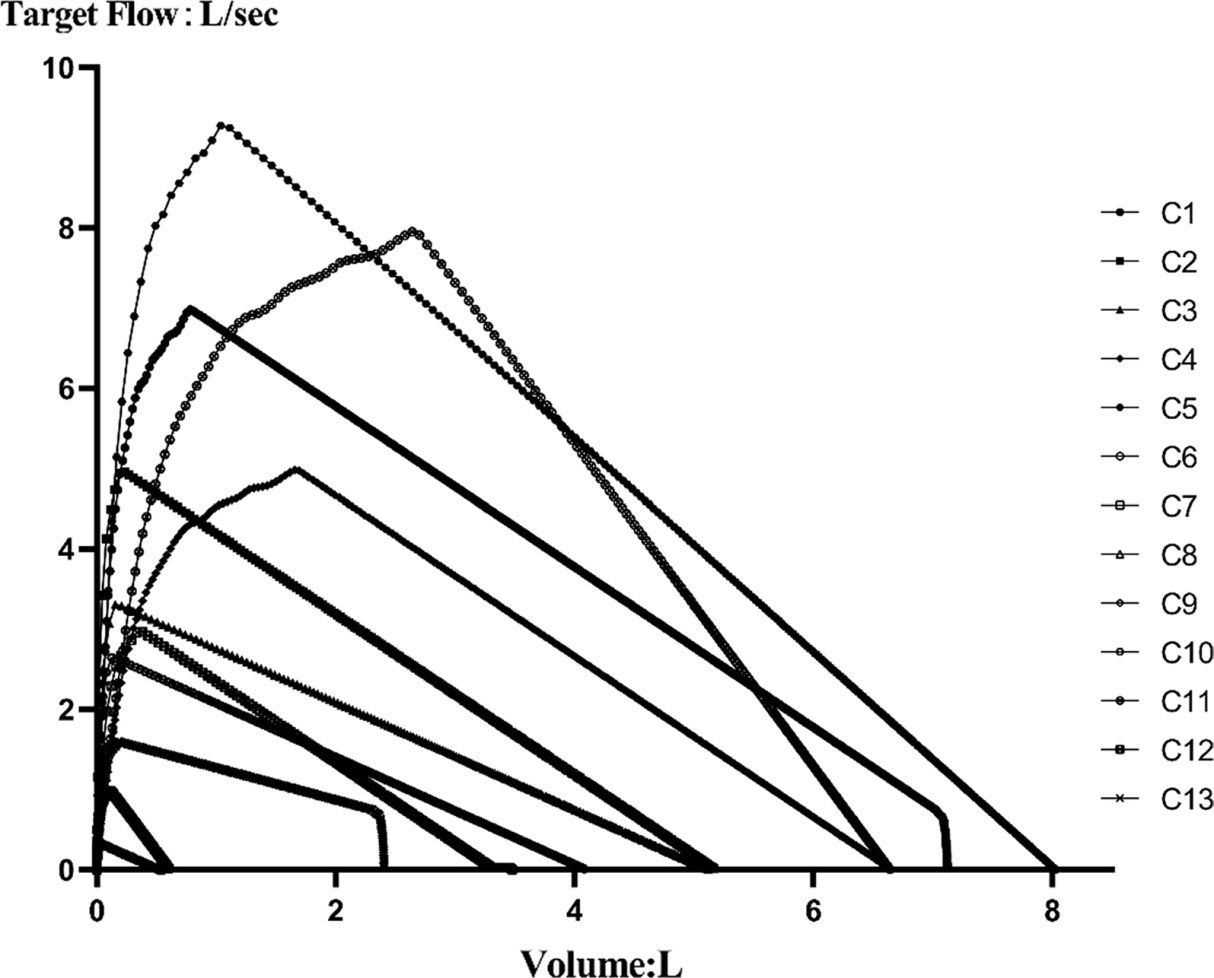
Flow-volume curve of the 13 waveforms

A total of 13 waveforms (C13 waveforms), defined in ISO 26782:2009, were selected to test the spirometers. These waveforms were derived from an exponential curve of volume as a function of time. According to the characteristics of pulmonary function in humans, the curve was used to generate different volume indexes, such as FEV_1_, FEV_6_ and FVC, through different expiratory time and volumes, as well as the characteristics of the start and end of forced exhalation [4]. We used C1-C13 to represent the 13 test waveforms and the flow-volume curves, derived from the simulator, were shown in Fig. 1.

### Methods and steps

Before testing, each spirometer and simulator was run 15–20 mins in advance at normal ambient temperature, pressure and relative humidity. Next, the spirometer was environmentally calibrated with a thermo-hygrometer, and volume calibration and linearity verification were performed with a 3 L standard calibration cylinder. We connected the spirometer to the simulator using a mouthpiece and rigid smoothbore coupling and noted the tightness of the connection. The simulator used ambient air to output air sources of defined testing waveforms C1-C11 to the 10 spirometers to measure FEV_1_, FEV_6_ and FVC 3 times for each waveform. The air pressure of each waveform was recorded both when the spirometers were connected and when they were not (including accessories and detachable parts), and peak impedance was measured when the volume output reached 1.0 L. For testing waveforms C12 and C13, air at 34℃ ± 2℃ and relative humidity greater than 90% was generated by the simulator to output the air sources; the same measurements were then performed as for waveforms C1-C11, but only for accuracy.

### Indicators of performance evaluation and their criteria

#### Evaluation indicators

(1) Accuracy (Verr): the difference in FEV_1_, FEV_6_, and FVC between the mean value from multiple measurements by the spirometer and the standard value from the simulator when processing the testing waveform.

1 $${\text{V}}_{{{\text{err}}}} = 1/3 \times (\sum\limits_{i = 1}^{3} {{\text{V}}_{{\text{i}}} } ) \, - {\text{V}}_{{{\text{ref}}}}$$

V_i_, measured volume; V_ref_, reference volume of the air source generated by the testing waveform.

(2) Repeatability (Vspan): the difference in FEV_1_, FEV_6_, FVC between the maximum and minimum value measured by the spirometer under the same waveform signal.

2 $${\text{Vspan}} = {\text{Vmax}} - {\text{Vmin}}$$

Vmax, maximum measured value; Vmin, minimum measured value.

(3) Linearity ($\epsilon n$): Verr from Formula 1 is used to calculate the linearity of adjacent waveforms among the C1-C11 testing waveform, which assesses whether the spirometer is linear across its measurement range.

3 $$\epsilon {\text{n}} = ({\text{Verr}}_{{\text{n}}} - {\text{Verr}}_{{{\text{n}} + 1}} ) \times 100/[0.5 \times ({\text{Vref}}_{{\text{n}}} + {\text{Vref}}_{{{\text{n}} + 1}} )]$$

Verr_n_, accuracy (from Formula 1) with the signal from waveform no. n. n, waveform number (1–11). Vref_n_, reference volume of the air source generated by waveform no. n.

(4) Airflow impedance (Zs): the impedance of each test waveform (in kPa/(L/s)), obtained by recording the peak pressure (kPa) and the corresponding flow (L/s) from the simulator when the simulator outputs 1 L of gas volume.

4 $${\text{Zs}} = {\text{ZT}} - {\text{ZA}}$$

ZT—total flow impedance of the system. ZA—flow impedance caused by other apparatuses measured without the spirometer.

#### Evaluation criteria

1. Accuracy: Fewer than 3 of the 13 testing waveform signals should have an average relative error of more than ± 3% of the reference value or ± 0.05 L, whichever is larger.
2. Repeatability: The repeatability of each of the C1-C11 testing waveform signals should not exceed  3% of the respective measured value or 0.05 L, whichever is larger.
3. Linearity: The number of calculated linearities Єn that cannot exceed 3% should be more than 15 among the C1-C11 testing waveform signals.
4. Impedance: The impedance of the spirometer (including its accessories and detachable parts) should not exceed 0.15 kPa/(L/s).

### Statistics

All the research data were analyzed statistically by Excel software, and the Bland–Altman diagram method of GraphPad Prism software was used to evaluate the consistency between the spirometer measurements and the standard values output by the simulator for different spirometers. If 95% of the points were within the range of consistency and acceptable error, the consistency of the tested instrument was considered good; otherwise, it was considered poor.

## Results

### Performance of the spirometers using the ISO 26782:2009 standard

In this study, 10 spirometers were tested by air sources generated by 13 waveform signals. Spirometers Nos. 1–3 were ultrasonic and Nos. 4–10 were differential pressure (Table 1). Spirometers 1 and 5 had no flow volume signal when tested by the C6 waveform signal, and spirometers 1, 5 and 10 had no flow volume signal when tested by the C8 waveform signal; the corresponding test data were thus missing and not included in the analysis. Finally, a total of 375 groups of data were obtained, all of which were analyzed for accuracy, repetition, linearity, impedance and so on. The results show that among the domestic spirometers, 3 pressure differential spirometers met the accuracy standard, while 2 pressure differential spirometers and 1 ultrasonic spirometer did not. Among the imported spirometers, 1 pressure differential spirometer and 1 ultrasonic spirometer met the accuracy standard (Additional file 1: Table S1). All ten tested spirometers passed the repeatability test (Additional file 1: Table S2). Regarding the linearity test, 2 domestic differential pressure spirometers and 1 imported differential spirometer did not meet the standard (Additional file 1: Table S3). Finally, the impedance of 3 domestic pressure differential spirometers was not up to the standard (Additional file 1: Table S4). Of the 10 spirometers, only spirometers 7, 8 and 9—2 domestic and 1 imported—met all performance standards, while the rest only partially met the standards. In addition, although Spirometer 1 met the standards for accuracy, repeatability, linearity, and impedance, it could not measure the air source generated by the C6 and C8 waveform signals, so it failed to fully meet the ISO standard (see Table 1 for details).

**Table 1:** Distribution of aspects of ISO 26782:2009 standard that ten spirometers meet

Spirometer number	Style	Accuracy	Repeatability	Linearity	Impedance	Standard meeting
*Domestic*						
2	Ultrasonic	×	√	√	√	Partially meeting
5	Differential pressure	×	√	×	×	Partially meeting
6	Differential pressure	√	√	√	×	Partially meeting
8	Differential pressure	√	√	√	√	Meeting
9	Differential pressure	√	√	√	√	Meeting
10	Differential pressure	×	√	×	×	Partially meeting
*Imported*						
1	Ultrasonic	√	√	√	√	Partially meeting
3	Ultrasonic	×	√	√	√	Partially meeting
4	Differential pressure	×	√	×	√	Partially meeting
7	Differential pressure	√	√	√	√	Meeting

Remarks: √ and X represent meeting and not meeting certain aspect of the standard respectively

### Analysis of the consistency of measured values across different spirometers

**Fig. 2:**
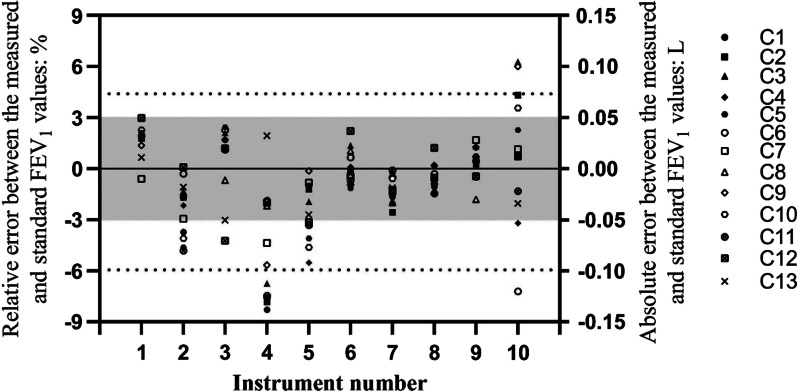
Bland–Altman diagram of error between the measured and standard FEV_1_ values for different spirometers. The dotted line represents the limit of consistency, and the shaded part represents the acceptable range

**Fig. 3:**
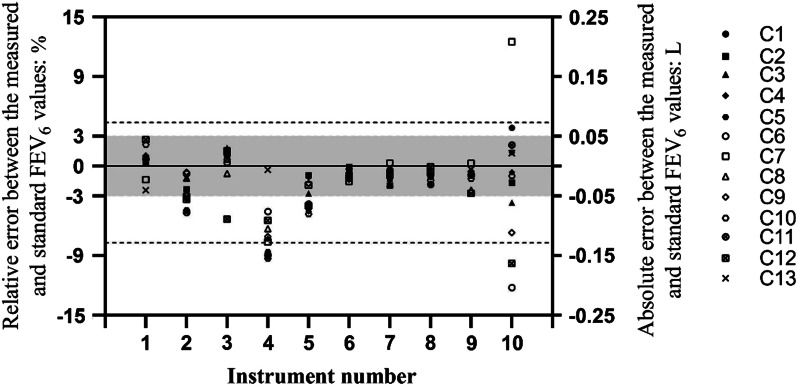
Bland–Altman diagram of error between the measured and standard FEV_6_ values for different spirometers. The dotted line represents the limit of consistency, and the shaded part represents the acceptable range

**Fig. 4:**
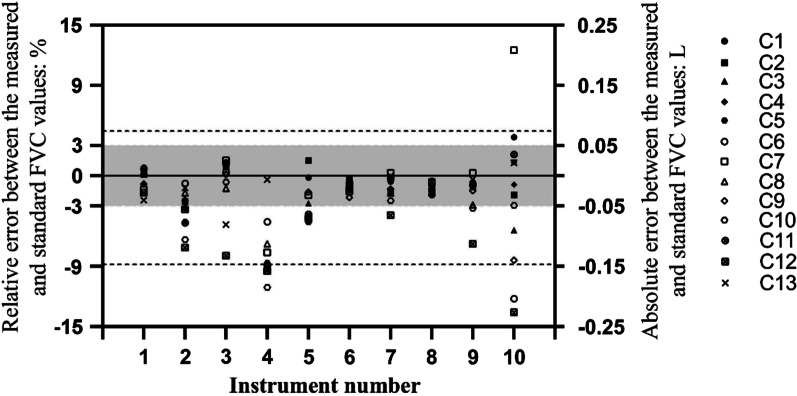
Bland–Altman diagram of error between the measured and standard FVC values for different spirometers. The dotted line represents the limit of consistency, and the shaded part represents the acceptable range

As seen from Figs. 2, 3 and 4, comparing the measured values of FEV_1_, FEV_6_ and FVC with the corresponding standard values from the simulator, the percentages of values within the range of consistency were 92.0% (115/125), 92.8% (116/125) and 93.6% (117/125), respectively, while 79.2% (99/125), 74.4% (93/125) and 70.4% (88/125) were within the range of acceptability.

In measuring FEV_1_, FEV_6_ and FVC, the performances of spirometers 1, 6 and 8 were within the ranges of both consistency and acceptability, while the performances of spirometers 2, 3, 5, 7 and 9 were only within the range of consistency or acceptability; the performances of spirometers 4 and 10 were out of the range of both consistency and acceptability.

## Discussion

At present, the quality evaluation of all clinical spirometers in China includes the comparison of their measurements in humans with control spirometers and analysis of their consistency. This method is often based on the assumption that the measured values of the control group are regarded as the gold standard, which are then compared with the measured values of the tested spirometers [5, 6]. However, in actuality, no spirometer is absolutely accurate, so this method is often unable to obtain reliable comparative data. In this study, an international standard simulator was used for verification and self-comparison. The servo motor of the simulator was precisely controlled by computer control software, driving the piston in the simulation cylinder to reciprocate expelling and intaking air, producing a gas whose volume can be accurately controlled for spirometer testing, and simulating different respiratory states. This method can be used to test the main technical indicators of a spirometer through a system driven by specific respiratory waveform signals. Therefore, the standard simulator can provide a reference for testing the accuracy, repeatability, and linearity of different spirometers.

Nelson [7], Jensen [8], and Schermer [9] had performed simulator-based quality testing and performance analysis of portable spirometers commonly used in different regions and found that there were deviations in the accuracy and repeatability of devices from different brands when using 24 volume-time curves [10] of the American Thoracic Society (ATS) standard. Except for the first 4 curves generated by mathematical formulas, the remaining 20 curves were all derived from the expiratory curves of real humans, including normal and abnormal expiratory curves. However, Lefebvre [11] believed that there was much redundance among the 24 waveforms, and expiratory curves involving steep rise time or low expiratory flow were not considered. In addition, the range of acceptable error for the 24 waveforms of the ATS standard is large, suggesting that it can only be used as the upper limit of the error, not as the standard [12]. In China, the national industry standard, the Specification for Calibration of Spirometer (JF1213-2008) [13], also requires regular spirometer quality inspection. However, as only the first edition has been released, its testing indicators and methods are relatively simple and quite different from those of the international standards and thus of little help in the development and quality control of domestic devices. In this study, 13 kinds of waveforms newly defined in ISO26782:2009 were used. In contrast to the 24 ATS waveforms, the 13 ISO waveforms are generated entirely by mathematical formulas by defining different volume and time constants based on main human characteristics as well as those of the start and end of forced exhalation. These 13 curves are smoother than the 24 ATS waveforms, and the number of tested curves is half that. The ATS guidelines—Standardization of Spirometry in 2019 [14]—recommend that the performance of spirometers should meet ISO standards. However, there have been no relevant studies on testing spirometers with the ISO 26782 standard.

The clinical application of spirometers is increasingly extensive, and the demand for scientific research at multiple centers is also increasing. To ensure accuracy in clinical examination and research data, it is necessary to achieve good spirometer technical status and quality control. This study found that after using the simulator to conduct the performance test, only 50% of the spirometers passed the accuracy test, including 3 domestic differential pressure spirometers, 1 imported ultrasonic spirometer and 1 imported differential pressure spirometer. Compared with the ultrasonic spirometers (33.33%, 1/3), the differential pressure spirometers had a higher accuracy test passing rate (57.14%, 4/7), but the results were not satisfactory. Sixty percent (3/5) of domestic differential pressure spirometers failed the impedance test, indicating that domestic manufacturers need to pay more attention to this problem. Spirometers 1, 5, and 10 could not produce results when tested by waveforms C6 or C8; the volume of the two curves is less than 1 L, with an FEV_1_ of only 0.26 L, and when such a low flow and volume of air passes through the sensor, some spirometers automatically default to the tidal respiration curve for those waveforms and thus ignore this signal without displaying the corresponding data. The manufacturer of these spirometers should readjust the algorithms and update the hardware to meet the standard and allow air sources of different flows and volumes to be tested. Our study has indicated that it is necessary to improve the accuracy of spirometers, as well as the linearity and impedance of the differential pressure spirometers. But the repeatability of all spirometers met the standard, indicating that the measurement has good stability.

Although only one instrument was tested for each brand of spirometer in this study, they were all new with guaranteed performance. Moreover, most spirometers may be updated quickly, so repeated testing over time to assess wear and error generation due to prolonged use was not performed. Therefore, the results of this study only provide a preliminary discussion on the performance of portable spirometers commonly used in China and do not serve as a unique reference for clinical selection.

## Conclusions

Through simulator testing, this study demonstrates certain deviations in the measurement of portable spirometers used clinically in our country, suggesting an urgent need to improve the quality management and update the quality inspection standard of spirometers in a timely manner. Spirometer manufacturers should use standard simulators for pre-factory quality inspection, and medical institutions should regularly test the instruments to ensure the accuracy of the measurements.

## Supplementary Information

**Additional file 1**. Test results of 10 different brands of spirometers

## Data Availability

All data generated or analyzed during this study are included in this published article.

## Abbreviations

ISO: International Organization for Standardization
FEV_1_: Forced expiratory volume in the first second
FEV_6_: Forced expiratory volume in 6 s
FVC: Forced vital capacity
ATS: American Thoracic Society
