# Supplementary material for: Technical performance analysis of different types of spirometers
Source: BMC Pulm Med. 2022 Jan 5;22:23. doi: 10.1186/s12890-021-01752-8 (PMC8734223; doi:10.1186/s12890-021-01752-8)
Supplement: Supplementary file 1 — Additional file 1. Test results of 10 different brands of spirometers [file 12890_2021_1752_MOESM1_ESM.docx]

**Supplementary tables (Inserted into Results 1.1)**

**Test results of 10 different brands of spirometers.**

Table S1. Accuracy of 10 different brands of spirometers

| **Measurement** | FEV_1_ | | | | | | | | | | FEV_6_ | | | | | | | | | | | FVC | | | | | | | | | |
| --- | --- | --- | --- | --- | --- | --- | --- | --- | --- | --- | --- | --- | --- | --- | --- | --- | --- | --- | --- | --- | --- | --- | --- | --- | --- | --- | --- | --- | --- | --- | --- |
| **Number**  **Waveform** | 1 | 2 | 3 | 4 | 5 | 6 | 7 | 8 | 9 | 10 | | 1 | 2 | 3 | 4 | 5 | 6 | 7 | 8 | 9 | 10 | 1 | 2 | 3 | 4 | 5 | 6 | 7 | 8 | 9 | 10 |
| C1 | 1.953 | -3.719 | 1.692 | -8.287 | -0.991 | -0.966 | -1.636 | -1.058 | 0.164 | 0.682 | | 0.839 | -3.230 | 1.360 | -8.694 | -1.043 | -0.388 | -0.572 | -1.077 | -0.618 | 3.868 | 0.839 | -3.230 | 1.360 | -8.694 | -0.171 | -0.388 | -0.572 | -1.077 | -0.618 | 3.868 |
| C2 | 1.817 | -1.666 | 1.226 | -7.844 | -1.189 | -0.070 | -2.564 | -0.644 | 0.429 | 4.323 | | 0.354 | -2.348 | 1.070 | -9.097 | -0.911 | -0.077 | -1.931 | -0.993 | -0.858 | -1.670 | 0.140 | -2.555 | 0.855 | -9.290 | 1.529 | -0.264 | -1.700 | -1.010 | -1.068 | -1.879 |
| C3 | 1.776 | -1.325 | 2.101 | -6.724 | -1.934 | 1.377 | -1.849 | 0.162 | 1.311 | 6.282 | | 1.088 | -1.200 | 1.845 | -7.258 | -2.747 | -0.703 | -1.248 | -0.869 | -0.404 | -3.658 | -0.729 | -2.976 | 1.342 | -8.798 | -2.708 | -1.179 | -1.373 | -1.218 | -1.120 | -5.389 |
| C4 | 2.124 | -2.149 | 1.212 | -7.666 | -5.515 | 0.106 | -1.991 | -0.837 | 0.656 | -3.183 | | 0.561 | -2.502 | 1.096 | -8.840 | -4.561 | -0.814 | -1.349 | -1.095 | -0.809 | -0.621 | 0.319 | -2.736 | 0.853 | -9.059 | -4.496 | -1.022 | -1.294 | -1.183 | -1.047 | -0.860 |
| C5 | 2.107 | -4.616 | 2.442 | -7.532 | -4.087 | -1.155 | -0.265 | -1.372 | 0.683 | 2.274 | | 0.421 | -4.507 | 1.382 | -8.587 | -4.343 | -1.321 | -0.530 | -1.807 | -0.980 | 1.399 | 0.396 | -4.531 | 1.357 | -8.609 | -4.367 | -1.345 | -0.434 | -1.831 | -1.005 | 1.374 |
| C6 | — | -0.005* | 0.019* | -0.031* | — | -0.008* | -0.008* | -0.005* | -0.008* | -0.120* | | — | -0.015* | 0.006* | -0.076* | — | 0.098* | -0.008* | -0.009* | -0.014* | -0.204* | — | -0.015* | 0.006* | -0.076* | — | 0.098* | -0.005* | -0.009* | -0.014 * | -0.204* |
| C7 | -0.010* | -0.049 * | 0.020* | -0.073* | -0.014* | -0.015* | -0.021* | -0.009* | 0.028* | 0.019* | | -1.351 | -3.335 | 1.533 | -7.621 | -1.905 | -1.526 | 0.276 | -0.697 | 0.283 | 12.504 | -1.351 | -3.335 | 1.533 | -7.621 | -1.905 | -1.526 | 0.276 | -0.697 | 0.283 | 12.504 |
| C8 | — | -0.023* | -0.011* | -0.036* | — | -0.012* | -0.033* | -0.023* | -0.030* | — | | — | -0.012* | -0.012* | -0.105* | — | 0.227* | -0.030* | -0.010* | -0.040* | — | — | -0.020* | -0.020* | -0.113* | — | 0.219* | -0.028* | -0.018* | -0.048* | — |
| C9 | 1.368 | -1.673 | 1.697 | -5.646 | -0.122 | 2.218 | -1.365 | 0.186 | 1.267 | 6.006 | | 1.022 | -0.605 | 1.168 | -7.083 | -1.741 | -1.309 | -1.185 | -0.937 | -0.539 | -6.699 | -0.804 | -2.401 | 0.130 | -8.762 | -1.602 | -2.133 | -1.304 | -1.262 | -1.465 | -8.385 |
| C10 | 2.275 | -4.089 | 2.268 | -7.579 | -4.619 | 0.665 | -0.584 | -0.899 | 0.696 | 3.555 | | 2.166 | -4.453 | 0.662 | -9.293 | -4.824 | -0.296 | -0.851 | -1.442 | -1.262 | -0.992 | 0.151 | -6.337 | -0.620 | -11.083 | -4.570 | -0.549 | -2.453 | -1.522 | -3.210 | -2.946 |
| C11 | 1.925 | -4.805 | 2.277 | -7.451 | -3.315 | -0.441 | -0.076 | -1.441 | 0.450 | -1.313 | | 0.712 | -4.654 | 1.197 | -8.930 | -3.817 | -0.703 | -0.415 | -1.843 | -0.904 | 2.140 | 0.712 | -4.654 | 1.197 | -8.930 | -3.817 | -0.703 | -0.219 | -1.843 | -0.904 | 2.140 |
| C12 | 2.974 | 0.101 | -4.218 | -1.987 | -3.121 | 1.056 | -1.346 | 1.219 | -0.448 | 0.724 | | 2.668 | -3.051 | -5.338 | -5.463 | -3.989 | -1.190 | -0.781 | -0.060 | -2.740 | -9.785 | -1.669 | -7.147 | -7.934 | -9.456 | -4.177 | -1.370 | -3.912 | -0.623 | -6.762 | -13.595 |
| C13 | 0.011* | -0.018* | -0.050* | 0.032* | -0.045* | -0.005* | -0.019* | -0.012* | 0.010* | -0.034* | | -2.423 | -1.187 | -5.284 | -0.352 | -4.029 | -0.942 | -0.139 | -1.061 | -0.333 | 1.321 | -2.423 | -1.187 | -4.849 | -0.352 | -4.029 | -0.942 | 0.000 | -1.061 | -0.333 | 1.321 |

Remarks: * represents the average absolute error (L); other values are relative errors (%). In some waveforms, if the volume of FEV_1_, FEV_6_ or FVC is less than 1.667 L, the accuracy should be expressed

in terms of absolute error, as its acceptable range is larger than that of relative error. — represents no data.

Table S2. Repeatability of 10 different brands spirometers

| **Measurement** | FEV_1_ | | | | | | | | | | FEV_6_ | | | | | | | | | | | FVC | | | | | | | | | | |
| --- | --- | --- | --- | --- | --- | --- | --- | --- | --- | --- | --- | --- | --- | --- | --- | --- | --- | --- | --- | --- | --- | --- | --- | --- | --- | --- | --- | --- | --- | --- | --- | --- |
| **Number**  **Waveform** | 1 | 2 | 3 | 4 | 5 | 6 | 7 | 8 | 9 | 10 | 1 | 2 | 3 | 4 | 5 | 6 | 7 | 8 | 9 | 10 | 1 | | 2 | 3 | 4 | 5 | 6 | 7 | 8 | 9 | 10 |  |
| C1 | 0 | 0.425 | 0.195 | 0.435 | 0.811 | 0.164 | 0 | 0.207 | 0 | 0.797 | 0.137 | 0.290 | 0.269 | 0 | 0.557 | 0.366 | 0.138 | 0.142 | 0 | 0.663 | 0.137 | | 0.290 | 0.269 | 0 | 0.276 | 0.366 | 0.138 | 0.142 | 0 | 0.663 |  |
| C2 | 0.295 | 0.624 | 0.588 | 0.649 | 0.304 | 0.332 | 0 | 0 | 0 | 0.576 | 0 | 0.793 | 0.372 | 0.830 | 0.383 | 1.007 | 0 | 0.195 | 0.195 | 1.543 | 0 | | 0.793 | 0.372 | 0.830 | 0.932 | 1.026 | 0.191 | 0.195 | 0.195 | 1.543 |  |
| C3 | 0 | 0.411 | 0.771 | 0 | 0.405 | 0.314 | 0.403 | 0 | 0.4 | 1.122 | 0.193 | 0.201 | 0.379 | 0.418 | 0.201 | 0.721 | 0.196 | 0.201 | 0.200 | 2.429 | 0.193 | | 0.201 | 0.374 | 0 | 0 | 1.008 | 0 | 0.198 | 0.375 | 2.429 |  |
| C4 | 0.236 | 0.503 | 0.236 | 0.519 | 0.255 | 0.123 | 0.245 | 0 | 0.244 | 0.249 | 0.147 | 0.927 | 0.289 | 0.161 | 0.310 | 0.213 | 0.149 | 0.152 | 0.152 | 1.635 | 0.147 | | 0.927 | 0.289 | 0.161 | 0.309 | 0.273 | 0.148 | 0.152 | 0.152 | 1.635 |  |
| C5 | 0 | 0.179 | 0.324 | 0.540 | 0.349 | 0.034 | 0.167 | 0.173 | 0 | 0 | 0 | 0.652 | 0.239 | 0.133 | 0 | 0.391 | 0.366 | 0.254 | 0.126 | 0.361 | 0 | | 0.652 | 0.239 | 0.133 | 0 | 0.391 | 0 | 0.254 | 0.126 | 0.361 |  |
| C6 | — | 0.020* | 0* | 0.019* | — | 0.004* | 0* | 0* | 0* | 0.010* | — | 0.030* | 0.010* | 0.019* | — | 0.006* | 0.010* | 0* | 0* | 0.010* | — | | 0.030* | 0.010* | 0.019* | — | 0.006* | 0* | 0* | 0* | 0.010* |  |
| C7 | 0.010* | 0* | 0.010* | 0.019* | 0* | 0.010* | 0* | 0* | 0* | 0.010* | 0 | 1.170 | 0 | 1.193 | 0 | 0.746 | 0 | 0.569 | 0 | 2.463 | 0 | | 1.170 | 0 | 1.193 | 0 | 0.746 | 0 | 0.569 | 0 | 2.463 |  |
| C8 | — | 0* | 0* | 0.019* | — | 0.001* | 0.010* | 0* | 0* | — | — | 0.010* | 0* | 0.019* | — | 0.015* | 0.029* | 0.010* | 0* | — | — | | 0.010* | 0* | 0.019* | — | 0.015* | 0* | 0.010* | 0* | — |  |
| C9 | 0 | 1.031 | 0.484 | 0 | 0 | 0.243 | 0.501 | 0.506 | 0 | 0.469 | 0.241 | 0.250 | 0.477 | 0.261 | 0 | 0.630 | 0.245 | 0.251 | 0.250 | 1.307 | 0.241 | | 0.250 | 0.237 | 0.261 | 0 | 0.823 | 0.241 | 0.247 | 0 | 1.307 |  |
| C10 | 0.208 | 0.454 | 0.206 | 0.459 | 0.447 | 0.213 | 0.426 | 0.219 | 0 | 1.030 | 0.152 | 0.663 | 0.153 | 0.170 | 0.163 | 0.540 | 0.623 | 0 | 0.160 | 2.040 | 0.152 | | 0.663 | 0 | 0.170 | 0.160 | 0.703 | 0.310 | 0.315 | 0.160 | 2.040 |  |
| C11 | 0 | 0.379 | 0.171 | 0.380 | 0.183 | 0.054 | 0 | 0 | 0 | 1.790 | 0 | 0.790 | 0.289 | 0.161 | 0.154 | 0.106 | 0.147 | 0.153 | 0 | 0.434 | 0 | | 0.790 | 0.289 | 0.161 | 0.154 | 0.106 | 0.147 | 0.153 | 0 | 0.434 |  |

Remarks: * represents the average absolute errors (L); other values are relative errors (%). In some waveforms, if the volume of FEV_1_, FEV_6_ or FVC is less than 1.667 L, the accuracy should be expressed

in terms of absolute error, as its acceptable range is larger than that of relative error. — represents no data.

Table S3. Linearity of 10 different brands of spirometers (%)

| **Measurement** | FEV_1_ | | | | | | | | | | FEV_6_ | | | | | | | | | | FVC | | | | | | | | | |
| --- | --- | --- | --- | --- | --- | --- | --- | --- | --- | --- | --- | --- | --- | --- | --- | --- | --- | --- | --- | --- | --- | --- | --- | --- | --- | --- | --- | --- | --- | --- |
| **Number**  **Waveform** | 1 | 2 | 3 | 4 | 5 | 6 | 7 | 8 | 9 | 10 | 1 | 2 | 3 | 4 | 5 | 6 | 7 | 8 | 9 | 10 | 1 | 2 | 3 | 4 | 5 | 6 | 7 | 8 | 9 | 10 |
| C1 | 0.887 | -3.126 | 1.048 | -3.659 | -0.236 | -1.099 | 0.090 | -0.753 | -0.147 | -2.643 | 0.675 | -1.769 | 0.676 | -2.423 | -0.442 | -0.385 | 0.961 | -0.412 | 0.005 | 5.887 | 0.853 | -1.588 | 0.854 | -2.242 | -1.485 | -0.228 | 0.769 | -0.396 | 0.184 | 6.061 |
| C2 | 0.540 | -0.757 | -0.413 | -3.143 | 0.312 | -1.268 | -1.328 | -0.873 | -0.640 | -0.487 | -0.715 | -1.197 | -0.734 | -2.065 | 1.786 | 0.615 | -0.727 | -0.150 | -0.471 | 1.914 | 0.866 | 0.388 | -0.474 | -0.597 | 4.230 | 0.906 | -0.345 | 0.194 | 0.039 | 3.468 |
| C3 | -1.302 | 1.674 | 0.078 | 4.464 | 5.404 | 0.920 | 1.081 | 1.164 | 0.174 | 8.707 | 0.30 | 1.812 | 0.343 | 3.802 | 2.822 | 0.320 | 0.460 | 0.497 | 0.572 | -2.446 | -0.994 | 0.504 | 0.203 | 2.587 | 2.726 | 0.130 | 0.268 | 0.278 | 0.210 | -3.715 |
| C4 | -0.747 | 3.689 | -1.890 | 2.612 | 0.307 | 1.454 | -1.317 | 0.934 | -0.269 | -5.293 | 0.046 | 2.669 | -0.521 | 1.398 | 0.626 | 0.709 | -0.641 | 0.987 | 0.341 | -2.094 | -0.145 | 2.475 | -0.711 | 1.205 | 0.701 | 0.545 | -0.698 | 0.931 | 0.150 | -2.282 |
| C5 | 3.766 | -8.315 | 3.896 | -12.848 | -6.365 | -1.883 | -0.223 | -2.361 | 1.504 | 7.928 | 1.179 | -8.092 | 2.433 | -14.197 | -6.429 | -2.100 | -0.801 | -3.150 | -1.498 | 7.321 | 1.138 | -8.136 | 2.386 | -14.24 | -6.468 | -2.146 | -0.697 | -3.196 | -1.544 | 7.272 |
| C6 | — | 4.989 | -0.137 | 4.704 | — | 0.867 | 1.388 | 0.454 | -4.082 | -15.753 | — | 3.925 | -1.786 | 4.931 | — | 0.980 | -1.080 | 0.280 | -1.598 | -35.742 | — | 3.925 | -1.786 | 4.931 | — | 0.980 | -0.807 | 0.280 | -1.598 | -35.742 |
| C7 | — | -3.417 | 4.081 | -4.806 | — | -0.484 | 1.598 | 1.840 | 7.622 | — | — | -3.368 | 3.445 | -2.628 | — | -1.307 | 3.032 | -0.174 | 3.920 | — | — | -2.662 | 4.128 | -1.925 | — | -0.608 | 2.869 | 0.521 | 4.601 | — |
| C8 | — | 0.887 | -3.956 | 6.677 | — | -4.926 | -0.524 | -2.364 | -4.876 | — | — | 0.176 | -2.612 | 7.923 | — | 1.788 | 0.779 | 1.202 | -0.806 | — | — | 3.024 | -1.113 | 10.633 | — | 2.909 | 1.090 | 1.440 | 0.518 | — |
| C9 | -2.362 | 4.718 | -2.155 | 7.216 | 6.391 | 0.409 | -0.002 | 1.370 | -0.213 | -1.368 | -1.851 | 4.968 | 0.101 | 5.840 | 4.538 | -0.658 | 0.117 | 1.032 | 1.123 | -4.002 | -0.809 | 5.880 | 0.859 | 6.735 | 4.341 | -0.987 | 1.985 | 0.880 | 2.785 | -2.919 |
| C10 | -0.044 | 1.550 | -0.436 | 1.282 | -0.560 | 1.085 | -0.447 | 0.762 | 0.138 | 4.657 | 1.381 | 0.432 | -0.582 | 0.098 | -0.789 | 0.432 | -0.403 | 0.484 | -0.304 | -3.161 | -0.574 | -1.514 | -1.826 | -1.845 | -0.624 | 0.173 | -2.193 | 0.372 | -2.243 | -5.073 |
| C11 | — | — | — | — | — | — | — | — | — | — | — | — | — | — | — | — | — | — | — | — | — | — | — | — | — | — | — | — | — | — |

Remarks: — represents no data.

Table S4. Impedance of 10 different brands of spirometers [kPa/(L/s)]

| **Number**  **Waveform** | 1 | 2 | 3 | 4 | 5 | 6 | 7 | 8 | 9 | 10 |
| --- | --- | --- | --- | --- | --- | --- | --- | --- | --- | --- |
| C1 | 0.030 | 0.032 | 0.048 | 0.080 | 0.283 | 0.260 | 0.069 | 0.110 | 0.055 | 0.272 |
| C2 | 0.021 | 0.021 | 0.031 | 0.066 | 0.206 | 0.164 | 0.057 | 0.079 | 0.044 | 0.289 |
| C3 | 0.013 | 0.014 | 0.020 | 0.055 | 0.165 | 0.108 | 0.051 | 0.070 | 0.035 | 0.320 |
| C4 | 0.022 | 0.021 | 0.032 | 0.065 | 0.211 | 0.165 | 0.060 | 0.080 | 0.043 | 0.284 |
| C5 | 0.040 | 0.041 | 0.064 | 0.090 | 0.324 | 0.336 | 0.082 | 0.150 | 0.063 | 0.285 |
| C6 | — | — | — | — | — | — | — | — | — | — |
| C7 | 0.009 | 0.009 | 0.010 | 0.046 | 0.105 | 0.080 | 0.042 | 0.040 | 0.021 | 0.388 |
| C8 | — | — | — | — | — | — | — | — | — | — |
| C9 | 0.011 | 0.011 | 0.017 | 0.052 | 0.144 | 0.082 | 0.046 | 0.063 | 0.032 | 0.338 |
| C10 | 0.034 | 0.035 | 0.053 | 0.084 | 0.297 | 0.344 | 0.074 | 0.122 | 0.057 | 0.282 |
| C11 | 0.031 | 0.031 | 0.047 | 0.077 | 0.259 | 0.232 | 0.069 | 0.101 | 0.053 | 0.275 |

Remarks: — represents no data.
